# Résultats de la plastie tubaire: étude tunisienne

**DOI:** 10.11604/pamj.2014.18.58.4128

**Published:** 2014-05-17

**Authors:** Kaouther Dimassi, Anissa Gharsa, Mohamed Badis Chanoufi, Ezzeddine Sfar, Dalenda Chelli

**Affiliations:** 1 Faculté de Médecine, Université de Tunis EL MANAR, Tunis, Tunisie; 2 Service de Gynécologie-Obstétrique A, Centre de Maternité, Tunis, Tunisie

**Keywords:** Infertilité, chirurgie de la reproduction, pathologie tubaire, Infertility, reproductive surgery, tubal disease

## Abstract

L'infertilité d'origine tubo-péritonéale est toujours d'actualité, sa fréquence reste stable, sinon croissante. La coelioscopie permet à la fois d'affirmer l'atteinte tubaire et de proposer un geste thérapeutique adapté. Le but de notre travail est d’évaluer les résultats de la chirurgie laparoscopique des pathologies tubaires en termes de grossesses obtenues. Il s'agit d'une étude rétrospective descriptive, analytique et longitudinale. Nous avons colligé les patientes suivies pour infertilité et opérées pour pathologies tubaires distales au service A du centre de maternité et de néonatologie de Tunis. Nous avons étudié les caractéristiques épidémiologiques et cliniques des patientes, les résultats de l'imagerie et détaillé les gestes chirurgicaux réalisés. Les résultats de la chirurgie tubaire distale étaient exprimés en termes de grossesses obtenues avec un recul minimal de 12 mois et maximal de 5 ans. 898 patientes étaient prises en charge dans le service pour une infertilité dont 52 patientes avaient répondu aux critères d'inclusion à l’étude. La sensibilité de l'hystérosalpingographie en matière de lésions tubaire était de 69% et la spécificité de 100%. Selon le score d'opérabilité tubaire distale, 23% des lésions étaient classées au stade 4 et 13.46% au stade 1. Le taux de grossesses spontanées était de 8.69%, soit 13% des fimbrioplasties et 4% des néosalpingostomie. Le délai de conception allait de 4 à 9 mois. Les antécédents ou stigmates d'infection pelvienne étaient retenus comme facteur de mauvais pronostic. Une sélection rigoureuse des patientes à partir des données de l'hystérographie et de la coelioscopie est indispensable afin de déterminer les patientes candidates à une chirurgie réparatrice ou à une fécondation in vitro

## Introduction

Les pathologies tubo-péritonéales représentent 26% des causes d'hypofertilité des couples et les hydrosalpinx sont retrouvés chez 10 à 15% des patientes suivies en procréation médicalement assistée (PMA) pour stérilité tubaire [1]. Elles font conséquence dans 80% des cas à des infections pelviennes d'origine gynécologique ou extra-gynécologique. Plusieurs explorations, notamment radiologiques, ont été proposées afin d’évaluer l'importance des lésions tubaires avec élaboration de différents scores [2, 3] permettant de définir avec plus de précision le pronostic ultérieur de fertilité. La coelioscopie permet à la fois d'affirmer l'atteinte tubaire et de proposer un geste thérapeutique adapté. En effet, elle permet une magnification de l'image autorisant une macrovision des lésions indispensable pour le diagnostic, le pronostic et le traitement. Le but de notre travail est d’évaluer les résultats de la chirurgie laparoscopique des pathologies tubaires en termes de grossesses obtenues.

## Méthodes

Nous avons mené une étude rétrospective descriptive, analytique et longitudinale sur une période de 5 ans allant de janvier 2007 à décembre 2011. Nous avons colligé les observations des patientes suivies pour infertilité et opérées pour pathologies tubaires distales au service A du centre de maternité et de néonatologie de Tunis.

Les critères d'inclusion étaient les suivants: - les patientes ayant une infertilité primaire ou secondaire; - un spermogramme du conjoint normal ou peu altéré; - un bilan hormonal des patientes correct (FSH12, E2 > 80); - le diagnostic d'infection utéroannexielle aigue était retenu; - les patientes n’étaient pas désireuses de grossesse.

Nous avons relevé à partir des dossiers d'hospitalisation les caractères épidémiologiques suivants: l’âge, la gestité, la parité, le type et la durée d'hypofertilité et les facteurs de risque de survenue d'infertilité tubo-péritonéale. Nous avons étayé les résultats des moyens diagnostiques (l’échographie, l'hystérographie et la coelioscopie diagnostique). A partir des comptes rendus opératoires, nous avons relevé les constatations per-opératoires ainsi que les gestes thérapeutiques réalisés. Pour la stadification des lésions tubaires distales nous avons adopté la classification de Donnez et al. [3]. En per-opératoire, l'aspect de la trompe et sa souplesse étaient évalués selon les classifications de Boer-Meisel [2] et Brossen [4] L’état de la muqueuse tubaire était établi par salpingoscopie ou tuboscopie. Elle consiste à introduire un endoscope à l′intérieur de la trompe à travers le pavillon pour explorer la lumière tubaire et la muqueuse qui tapisse la trompe de l′intérieur jusqu′à la jonction isthmo-ampullaire. Toutes les données étaient recueillies sur Epi info 6, et l'analyse statistique était réalisée par le logiciel SPSS 15.0. Les patientes ont été contactées par téléphone pour évaluer les résultats de la chirurgie tubaire distale en termes de grossesses obtenues spontanément ou après PMA avec un recul minimal de 12 mois et maximal de 5 ans.

## Résultats

Durant la période d’étude, 898 patientes étaient prises en charge dans le service pour une infertilité. Parmi elles, 52 patientes avaient répondu aux critères d'inclusion à l’étude (6%). L’âge moyen des patientes était de 34 ans (allant de 21 à 48 ans). 12 patientes étaient âgées de plus de 40 ans. La gestité moyenne était de 2 (allant de 0 à 5) avec 17 (33%) nulligestes. 23 patientes (44%) étaient nullipares dont 9 (39%) étaient âgées de plus de 35 ans. 35 patientes avaient une infertilité secondaire. La durée de l'infertilité moyenne était de 48 mois (extrêmes allant de 6 à 192 mois). 26 patientes (50%) avaient un antécédent de chirurgie pelvienne (5 cas de chirurgie digestive, 6 cas de chirurgie tubaire, 5 cas de chirurgie ovarienne et 10 cas de chirurgie utérine). 17 patientes (33%) avaient eu une contraception mécanique par dispositif intra-utérin (DIU). 18 patientes (35%) avaient des antécédents d'infections génitales hautes traitées médicalement dont la plupart faisaient suite à un accouchement, des manoeuvres endo-utérines ou à l'utilisation d'un DIU. Le germe le plus souvent incriminé était le Chlamydiae Trachomatis. Nous avons noté que la sérologie chlamydiae était positive chez trois patientes sans antécédent notable d'infection génitale haute. Deux patientes avaient des antécédents de tuberculose (TBC) (1 cas de TBC péritonéale et 1 cas de TBC pulmonaire). La recherche du bacille de Koch dans les urines était positive dans les deux cas.

L’échographie systématique était normale dans 60% des cas et avait évoqué un kyste ovarien chez 9 patientes. L'hystérosalpingographie était pratiquée dans 67% des cas. Chez 29 patientes, les lésions tubaires étaient unilatérales et dans 6 cas, l'atteinte était bilatérale. Nous avons relevé 47 trompes pathologiques la répartition des lésions selon Donnez et al. [3] est détaillée dans le Tableau 1. La sensibilité de l'hystérosalpingographie en matière de lésions tubaire distale était de 69% et la spécificité de 100%. Chez trois patientes avec des images latéro-utérines à l’échographie, un complément d'exploration par tomodensitométrie pelvienne était demandé concluant à un hydrosalpinx dans 2 cas. Une seule fertiloscopie était pratiquée chez une patiente avec un antécédent de TBC. L'examen a montré des adhérences multiples et un complément de coelioscopie a été réalisé.

**Tableau 1:** Répartition des lésions tubaires selon la classification de Donnez et al.[3]

Stade de l'atteinte tubaire	Nombre de cas
Stade 1:	0
Stade 2: obstruction tubaire distale.	7
Stade 3: dilatation de moins de 3cm, plis conservés.	18
Stade 4: hydrosalpinx simple.	14
Stade 5: paroi épaissie, disparition des plis	8

Au cours de la coelioscopie, nous avons objectivé des adhérences pelviennes associées ou non au syndrome de Fitz Hugh Curtis chez 46 patientes (88%). Les lésions tubaires étaient bilatérales dans 16 cas: 14 cas d'hydrosalpinx bilatéraux avec une taille allant de 2 à 8 cm, un cas de phimosis droit avec une trompe gauche totalement adhérente à l'ovaire homolatéral et un cas d'hydrosalpinx gauche et un phimosis controlatéral.

L’épreuve de perméabilité tubaire était négative chez toutes les patientes.

La paroi tubaire était jugée épaisse dans 22% des cas. L'exploration des muqueuses tubaires nous a permis de relever la présence des plis muqueux pour 17 trompes pathologiques (25%), des plis diminués pour 32 trompes (47%) et des plis absents pour 12 trompes (18%). Pour 7 trompes, l’évaluation était jugée difficile.

Nous avons trouvés chez deux cas des lésions d'endométrioses avec des adhérences sévères. Selon le score français d'opérabilité tubaire distale [5], 12 (23%) lésions étaient classées au stade 4 et seulement 7 (13.46%) au stade 1. La décision thérapeutique avait pris en considération le stade de la lésion mais aussi l’âge de la patiente, ses conditions socio-économiques la couverture sociale et l’éventuel refus de passage en PMA. Les gestes opératoires réalisés sont détaillés dans le Tableau 2.

**Tableau 2:** Différentes conduites thérapeutiques réalisées dans l’étude

Conduite thérapeutique	Nombre de lésions
Abstention	01
Adhésiolyse simple	14
Fimbrioplastie	15
Néosalpingostomie	27
Salpingectomie	11
TOTAL	68

Dans 7 cas, l'adhésiolyse seule avait permis de rétablir la perméabilité tubaire, ailleurs le geste a été complété par une fimbioplastie. La néosalpingostomie était réalisée pour les lésions de stade 1 ou 2 et 3 avec une muqueuse tubaire de bonne qualité, une obstruction proximale associée ou encore en cas de refus du traitement radical par la patiente. Pour les lésions classées au stade 4, la conduite était une salpingectomie totale à l'exception d'une patiente âgée de 48 ans ne pouvant ainsi pas bénéficier de la PMA. Les indications du traitement radical sont détaillées dans le Tableau 3. Le taux de reperméabilisation après néosalpingostomie était de 67%.

**Tableau 3:** Indications de la salpingectomie totale

Indication de la salpingectomie	Nombre de patientes
Torsion tubaire	01
Hydrosalpinx de taille supérieure à 7 cm	05
Récidive après plastie tubaire	02
Adhérences péritonéales multiples et paroi tubaire épaissie	03
TOTAL	11

Nous n'avons pas relevé de complications hémorragiques. Une patiente a présenté un pyosalpinx en post opératoire et a nécessité une reprise chirurgicale avec salpingectomie totale. Un suivi minimum de 12 mois a été possible pour toutes les patientes. Les résultats du suivi sont détaillés dans le Tableau 4.

**Tableau 4:** Résultats du suivi des patientes de l’étude

Evènement	Nombre de patientes
Adénocarcinome de l'endomètre	01
Décès	01
Grossesse spontanée	04
Grossesse extra-utérine	02
Grossesse après PMA	02
Infertilité persistante	44
Total	52

Une patiente âgée de 40 ans avait consulté 01 an après la coelioscopie pour des métrorragies. Les explorations avaient retenu le diagnostic d'adénocarcinome de l'endomètre et la conduite a été une chirurgie radicale. Une patiente était décédée par accident de la voie publique, 14 mois après l'opération, sans obtention de grossesse dans cet intervalle. Parmi les 50 patientes restantes, une patiente avait eu une salpingectomie bilatérale et trois patientes avaient eu une salpingectomie unilatérale avec obstruction proximale controlatérale. Ainsi, sur les 46 patientes avec plastie tubaire ou salpingectomie totale unilatérale, quatre avaient obtenu des grossesses spontanées soit 13% des fimbrioplasties et 4% des néosalpingostomie. Le délai de conception allait de 4 à 9 mois. Aucune de ces quatre patientes n'avait d'antécédent infectieux ou de stigmates d'infection en per-opératoire.

Parmi les 50 patientes, seulement 8 avaient eu une tentative de PMA par fécondation in vitro, toutes soldées par un échec.

Les délais de conception variaient de 4 à 9 mois. Chaque patiente a eu une seule grossesse. Les 4 patientes qui ont conçu spontanément n'ont ni d'antécédent infectieux ni de stigmates d'infection en per opératoire. Parmi les 50 patientes, huit patientes ont eu une tentative de procréation médicalement assistée. Nous avons trouvé un échec de deux tentatives de FIV pour deux patientes et 6 patientes ont eu un échec d'une tentative de FIV.

En discutant avec les patientes, nous avons remarqué qu'un grand nombre d'entre elles n'avaient pas les moyens pour un passage en PMA, certains couples n'avaient pas d'idée sur la PMA et d'autres refusaient concept.

## Discussion

L'infertilité d'origine tubo-péritonéale est toujours d'actualité, sa fréquence reste stable, sinon croissante. Ainsi, les pathologies tubo-péritonéales, cause d'infertilité de 26% des couples infertiles [1], regroupent les salpingopathies distales, proximales, les malformations, les adhérences et l'endométriose. L'origine infectieuse est la plus fréquente donnant plus de 80% des lésions tubo-péritonéales [6]. Dans notre groupe, les patientes ayant seulement une stérilité tubaire distale, sans autres facteurs d'infertilité, représentaient 6% des couples infertiles. Ce taux est potentiellement réduit en comparaison à d'autres séries lié au plus faible taux d'infections sexuellement transmissibles (IST). Le diagnostic des pathologies tubaires distales est suspecté devant l'association des signes échographiques et hystérosalpingographiques et confirmé par la coelioscopie.

L'hystérosalpingographie fait partie du bilan d'exploration du bilan d'infertilité. La méta-analyse de Swart et al pour le diagnostic d'hydrosalpinx a montré que l'hystérosalpingographie avait une sensibilité de 65% et une spécificité de 83% par comparaison avec la coelioscopie [7, 8]. Dans notre étude, nous avons trouvé une sensibilité de 69% et une spécificité de 100%.

L’échographie pelvienne et surtout par voie endovaginale ne montre que les hydrosalpinx distendus en permanence et les plus volumineux. Selon Atri et al [9], l’échographie a une sensibilité de 100% et une spécificité faible de 34%. Mais dans nos résultats, nous avons constaté une sensibilité de seulement de 30%.

La pratique de plus en plus diffusée de l’échographie tridimensionnelle avec possibilité de reconstruction spatiale permet de faire la distinction entre un hydrosalpinx et un kyste ovarien et de poser le diagnostic de quasi-certitude [10].

L'IRM et le scanner sont des examens de deuxième intention. Ils ont un intérêt en cas de diagnostic difficile ou un doute diagnostique. Dans sa forme volumineuse, l'hydrosalpinx peut être confondu avec une tumeur multiloculaire et la recherche des septa incomplets aide pour le diagnostic différentiel. Dans notre étude, le scanner a permit de redresser le diagnostic d'hydrosalpinx chez deux patientes.

La c'lioscopie est la seule exploration qui permet de poser le diagnostic de salpingopathies distales avec certitude. Elle permet dans le même temps d’évaluer l’état du pelvis à la recherche d'adhérences et de réaliser une épreuve de perméabilité tubaire. On peut apprécier l’état de la trompe pathologique en décrivant la souplesse de la paroi, la taille de la trompe par son diamètre et la souplesse du pavillon. Lors de la coelioscopie, une salpingoscopie (la tuboscopie) consiste à introduire un endoscope à l′intérieur de la trompe à travers le pavillon pour explorer la lumière tubaire et la muqueuse qui tapisse la trompe de l′intérieur jusqu′à la jonction isthmo-ampullaire, ce qui correspond à peu près aux deux tiers distaux de la trompe. Elle peut être réalisée pour évaluer l’état de la muqueuse tubaire et de révéler un nombre significatif [11, 12] de lésions tubaires non détectées par l'hystérosalpingographie. Elle peut se faire par voie haute lors de la coelioscopie ou par voie vaginale lors de la fertiloscopie [13].

Les éléments pronostiques sont essentiellement relevés du bilan per opératoire et sont les suivants: -une paroi rigide, épaisse et fibreuse de l'hydrosalpinx [14, 15] - la taille de l'hydrosalpinx, avec un diamètre au-delà de 2 cm [16, 17] - une diminution des plis muqueux - la présence d'adhérences intra-luminales [18] - la présence de plus de 50% de surface muqueuse atteinte [17] - la présence d'adhérences péri hépatiques [17] - le caractère bilatéral des lésions.

Deux types de prise en charge permettent à ces patients des chances de conception: l'assistance médicale à la procréation et la chirurgie tubaire distale. Cette dernière dont l'objectif est la reconstitution d'une trompe fonctionnelle, permet d'espérer des taux de grossesse intra-utérine aux alentours de 30% [18–20].

Dans notre étude, le taux de conception après plastie tubaire était faible de 8.69% des cas. Ce ci peut être expliqué par le nombre élevé de lésions tubaires avancées (23% stade 4 contre seulement 13% au stade 1 selon le score français d'opérabilité tubaire distale [5]) Par ailleurs, nous avons noté que l'antécédent d'infection génitale était un élément de mauvais pronostic. Les patientes qui sont arrivées à concevoir n'avaient ni d'antécédent étiqueté d'infection génitale ni des sérologies positives à Chlamydiae ni des stigmates d'infection à la coelioscopie.

De nombreux travaux [21–23] ont souligné le rôle délétère des hydrosalpinx sur les chances d'implantation embryonnaire et les taux de grossesse [24, 25]. Ainsi, l'abstention thérapeutique ne semble actuellement pas légitime.

La chirurgie commence par une adhésiolyse. L'adhésiolyse coelioscopique a des limites et l'opportunité d'une adhésiolyse parfois longue et complexe devrait toujours tenir compte des scores d’évaluation.

Plusieurs publications confirment la corrélation du score initialement fixé lors de la coelioscopie aux chances de succès de l'intervention en termes de fertilité ultérieure. Alborzi et Oeslner [21, 25] rapportent plus de 50% de grossesses spontanées après adhésiolyse d'adhérences fines mais un taux inférieur à 30% en cas d'adhérences denses. Dans les cas d'atteintes sévères, lorsque les adhérences sont denses, vascularisées et/ou extensives réalisant à l'extrême un tableau de « pelvis gelé », la prise en charge en AMP d'emblée se justifie compte tenu du risque opératoire et du bénéfice très improbable de ce geste [22]. Ainsi, l'adhésiolyse itérative, lorsqu'elle s'accompagne d'une « escalade adhérentielle » doit être proscrite. Dans notre étude nous n'avons pas observé de grossesse spontannée après adhésiolyse seule. Il faut souligner ici que le risque de grossesse ectopique ultérieur ne doit pas interférer dans le choix entre chirurgie et AMP.

La fimbrioplastie consiste en la réfection de l'anatomie normale du pavillon à partir de l'ancien ostium tubaire à lumière diminuée. Si les franges muqueuses du pavillon sont agglutinées, il suffit le plus souvent de dilater la sténose en introduisant la pince fine atraumatique, les mors fermés, puis d'ouvrir ces derniers doucement. Si cela ne suffit pas, les bandes de tissus sclérosés seront incisées aux ciseaux. Une épreuve au bleu termine l'intervention afin de contrôler la perméabilité tubaire [1]

La néosalpingostomie consiste en la réalisation d'un ostium tubaire de novo au niveau de la zone de l'ancien orifice. Après avoir effectué une adhésiolyse complète, un bilan tubaire selon le score tubaire d'opérabilité français [5] permet de définir un bilan d'opérabilité tubaire. Ainsi, les stades tubaires I et II peuvent bénéficier d'une chirurgie tubaire distale. En ce qui concerne les stades III, la décision est prise en fonction de l’état de l'endosalpinx évalué par salpingoscopie. Les stades IV sont une contre-indication formelle à la chirurgie tubaire distale.

La perméabilité tubaire est le reflet de l'aspect purement technique de la réussite de l'intervention. Affirmée par hystérosalpingographie ou par coelioscopie, 70 à 85% des trompes ont retrouvé une perméabilité, tous stades tubaires confondus. Dans les stades tubaires I et II, les résultats attendus se situent aux environs de 90-100% [25, 26]. Les taux de reperméabilisation sont d'autant meilleurs que le score tubaire l’était en per opératoire. Dans notre étude, le taux de reperméabilisation après néosalpingostomie était de 67%. Dans la littérature, les taux de réobstruction complète avoisinent les 4% et la reprise chirurgicale des réobstructions montre de mauvais résultats avec des taux de grossesse inférieurs à 10% [27, 28]. En effet, les taux de reperméabilisation sont excellents [29] et malgré une perméabilité tubaire correcte, les chances de grossesse intra-utérine ne sont pas identiques à une population de patientes à trompes saines. Les études histologiques de parois tubaires à distance d'une reperméabilisation retrouvent une déciliation persistante au niveau de l’épithélium tubaire [30], confirmant le caractère chronique et irréversible des lésions cellulaires. Ainsi, la restitution de la perméabilité tubaire est nécessaire mais pas suffisante dans le traitement des obstructions tubaires.

En matière de fertilité spontanée après plastie tubaire, les deux techniques (néosalpingostomie et fimbrioplastie) sont souvent assimilées et il est parfois difficile d'appréhender les résultats de l'une ou l'autre prise en charge chirurgicale. Les taux de grossesse pour la néosalpingostomie sont situés entre 10 et 33% [18, 26, 27, 31–33] et pour la fimbrioplastie entre 20 et 60% [18, 32, 34–37]. Dans notre étude, les taux étaient respectivement de 13% en cas de fimbrioplasties et 4% en cas de néosalpingostomie. Les résultats de la chirurgie tubaire distale sont bien corrélés au score tubaire déterminé par les conclusions de l'hystérographie et de la c'lioscopie [1]. Ainsi, les stades I et II constituent une excellente indication à la néosalpingostomie. Les stades III tubaires sont associés à des taux de grossesse intra-utérine entre 0 et 10% avec des taux de grossesse extra utérine identiques [20, 32, 36]. Devant une lésion de stade IV, les chances de grossesses intra-utérine et extra-utérine sont inférieures à 5%. Il s'agit dans la plupart des cas d'hydrosalpinx à parois épaisses et rigides. Le caractère rigide rend la néosalpingostomie techniquement difficile avec une paroi difficilement manipulable. La trompe a non seulement perdu son rôle de transporteur de l'ovocyte, mais l'altération sévère du pavillon et de la muqueuse ostéale ne lui permet plus de capter l'ovocyte, expliquant le faible taux de grossesses aussi bien intra-utérine qu'extra-utérine.

La majorité des grossesses spontannées intra-utérines surviennent au cours de la première année avec une chute de la probabilité de conception au fur et à mesure que l'on s’éloigne de l'intervention [18, 26, 33]. Les taux cumulés de grossesse intra-utérine à 1 an se situent aux alentours de 25% [18, 27]. Dans notre étude, le taux de grossesse spontanée était de seulement de 8.69% des cas. Ce ci peut être expliqué par le nombre élevé de lésions tubaires avancées.

La salpingectomie a fait la preuve de son efficacité dans l'amélioration des résultats de la FIV puisqu'elle permet aussi bien une augmentation des taux de grossesse que des naissances vivantes à terme [38]. Dans les cas où une FIV est d'emblée requise (en cas de facteur masculin associé par exemple), l'indication de la salpingectomie ne pose pas question. Toutefois la difficulté dans le cas des hydrosalpinx réside dans la distinction des patientes pouvant bénéficier d'une plastie tubaire distale et celles pour lesquelles la salpingectomie est préférable. Le score tubaire distal d'opérabilité répond à cette interrogation [5]. Compte tenu des mauvais résultats de la salpingostomie distale en cas de stade IV, la prise en charge de ces patientes doit s'orienter vers la salpingectomie première puis la prise en charge en FIV. Dans notre étude, pour les lésions classées au stade 4, la conduite était systématiquement une salpingectomie totale à l'exception d'une patiente âgée de 48 ans ne pouvant ainsi pas bénéficier de la PMA. La question est plus délicate en ce qui concerne le stade III où l’état de la muqueuse tubaire devrait emporter la décision. En l'absence de plissement muqueux persistant, il existe des raisons de penser que le caractère non fonctionnel de la trompe est définitif quel que soit le résultat anatomique du traitement de l'obstruction distale. Toutefois, bien que des recommandations officielles existent concernant la prise en charge des hydrosalpinx [39], il est regrettable d'observer que moins de la moitié des centres pratiquant la FIV les appliquent [40].

Enfin, nous n'avons pas un bon recul en procréation médicalement assistée. Aucune grossesse sur dix tentatives de fécondation in vitro réalisées chez huit patientes. Le nombre des patientes était réduit pour conclure l'effet bénéfique de la salpingectomie pour hydrosalpinx avant une procréation médicalement assistée bien que ça était démontré dans la littérature [41, 42].

## Conclusion

Une sélection rigoureuse des patientes à partir des données de l'hystérographie et de la coelioscopie est indispensable afin de déterminer les patientes candidates à une chirurgie réparatrice ou à une fécondation in vitro. L’évaluation de la trompe et notamment de la muqueuse tubaire lors de la coelioscopie est un des éléments majeurs lors de la décision thérapeutique et dans le pronostic de grossesse spontanée.
